# Risk perceptions of COVID-19 in Beijing: a cross-sectional study

**DOI:** 10.3389/fpsyg.2024.1294765

**Published:** 2024-02-07

**Authors:** Qing Liu, Yiyang Tan, Zheng Zhu, Jiawei Zhang, Yaqun Fu, Quan Wang, Zhijie Nie, Li Yang, Xiaoguang Li

**Affiliations:** ^1^General Practice Department, Second Outpatient Section, Peking University Third Hospital, Beijing, China; ^2^Department of Health Policy and Management, Peking University School of Public Health, Beijing, China; ^3^Department of Infectious Diseases, Peking University Third Hospital, Beijing, China

**Keywords:** COVID-19, risk perception, determinants, cross-sectional study, pandemic

## Abstract

**Background:**

The Chinese government has ended the “dynamic zero-COVID” policy, and residents are now living together with the SARS-CoV-2 virus. Only a limited number of studies have investigated the specific content and structure of COVID-19-related risk perceptions, as well as their underlying determinants. This study measured the residents’ risk perception of COVID-19 and analyzed the predictors of RP.

**Methods:**

We conducted a comprehensive questionnaire-based survey among residents mostly in Beijing, using a specially designed scale consisting of 11 items to accurately measure COVID-19 risk perceptions. We then utilized multiple linear regression analysis to investigate the factors associated with risk perceptions.

**Results:**

A total of 60,039 residents participated in the survey. Our study reveals that COVID-19-related worries are significantly influenced by other dimensions of RP (*p* < 0.001), except for perceived society’s control of the epidemic. Several experiential and socio-demographic factors, including gender, educational level, and infectious experience, are notably correlated with all dimensions of risk perceptions of COVID-19.

**Conclusion:**

This study evaluates the specific content and structure of COVID-19-related risk perceptions, as well as their determinants. It is essential to understand the risk perceptions and health-protective behaviors of residents with diverse educational levels, incomes, and medical histories.

## 1 Introduction

### 1.1 Background

COVID-19, also known as the coronavirus disease 2019, is a highly contagious respiratory illness caused by the SARS-CoV-2 virus. It was first identified in December 2019 and has since spread rapidly, becoming a global pandemic that affects billions of people worldwide. As of May 21st, 2023, over 766 million confirmed cases and over 6.9 million deaths have been reported globally, which is revealed by WHO in the latest edition of *COVID-19 Weekly Epidemiological Update*. The disease is primarily spread through respiratory droplets when an infected person talks, coughs, or sneezes, and also by exposure to surfaces contaminated with the virus. COVID-19 can cause a range of symptoms, from mild to severe and even fatal, including fever, cough, shortness of breath, fatigue, and loss of taste or smell. Older adults are at higher risk of severe disease and death than younger ones (Chen et al., 2021) and evidence suggests that there are remarkable racial and ethnic disparities in SARS-CoV-2 infections and COVID-19 hospitalizations (Mackey et al., 2020). In response to the pandemic, the World Health Organization declared the outbreak of COVID-19 a “public health emergency of international concern” (PHEIC), on the historical date of January 30th, 2020.

To prevent the spread of COVID-19, health-protective behaviors, such as social distancing, wearing masks, and frequent hand washing are highly recommended. Besides providing healthcare information, many countries also adopt governmental measures which can be commonly divided into elimination (known as “zero-COVID”) and mitigation (known as “flattening the curve”) (Thunström et al., 2020; Al-Mustapha et al., 2021). During the COVID-19 pandemic, the Chinese government is specifically committed to the national “dynamic zero-COVID” strategy and it has turned out to be an enormous success (Burki, 2022). Yet, the Chinese authorities cannot help but think about the adjustment of the zero-COVID policy, considering the mighty power of the Omicron variant and unignorable socioeconomic issues (Chen and Chen, 2022). On December 7th, 2022, China officially announced the “new 10 prevention and control measures” after enforcing the elimination policy for more than two years. Since then, the Omicron pandemic has spread swiftly in major cities in China, including Beijing, the capital where the predominant Omicron BF.7 has put significant pressure on the healthcare system. By December 22nd, it is estimated that the Omicron outbreak had peaked in Beijing, with 76% of the Beijing residents infected (Leung et al., 2023). On 5 May 2023, Tedros Adhanom Ghebreyesus, the World Health Organization (WHO) Director-General, eventually declared that COVID-19 “is now an established and ongoing health issue which no longer constitutes a PHEIC.”

### 1.2 Contagion: risk perception during and after pandemics

Research on risk perception began in the 1940s when Gilbert White published his pioneering paper, *Human Adjustment to Floods*. White redefined how human responses to hazards should be studied (White, 1942) and found that personal experience with floods directly affected people’s behavior when they were under a similar threat again (Macdonald et al., 2012). In other words, a brand new way to research risk and multihazard environments has been brought out. In 1969, Chauncey Starr discovered systematic relationship between the acceptance of technology risks and perceptions of costs and benefits, based on a revealed preference approach (Starr, 1969). Over the following decades, risk perception research evolved into psychological experiments and public surveys in which individual perceptions could be assessed with the help of several theories and approaches (Kellens et al., 2013). As such, studies have shown that risk perception is a subjective mental construction that is influenced by cognitive, experiential, and socio-cultural factors (De Dominicis et al., 2015; van der Linden, 2015). Even though higher levels of risk perception may have a positive impact on improving individuals’ behaviors when faced with environmental risks, it is suggested that RPs need to be accompanied by coping appraisal for a positive response (Bubeck et al., 2012). Among victims of natural disasters, concern about risk is notably associated with psychological stress, with possible feedback from pre-adopted mitigation measures on RPs (Suzuki et al., 2015).

Since the COVID-19 pandemic outbursted in 2019, research has demonstrated that perceived risk and knowledge are influential factors which would shape individuals’ engagement in health-protective behaviors and affect their intentions toward vaccination, as well as their mental status (Faasse and Newby, 2020; Motta Zanin et al., 2020). Approximately 43.6% of the participants perceived themselves at a high risk of exposure to COVID-19, while 50% considered the disease to be serious (Honarvar et al., 2020). Additionally, older adults are more likely to associate a higher risk of death with COVID-19 infection (Bruine De Bruin, 2021). Experts in work and organizational psychology have been focusing on the perception of COVID-related risks and mental well-being as well. The perception of worker safety can be articulated through four distinct dimensions that reflect the organization’s capability amidst the COVID-19 pandemic, namely “situational awareness, capacity to communicate and make decisions effectively and efficiently, and the capacity to recognize additional mental and physical fatigue (Flin et al., 2013).”

Nowadays, the COVID-19 pandemic has entered a new phase characterized by fluctuations in transmission rates but with lower mortality rates. This study utilized a standardized scale to measure COVID-19 risk perception, while also collecting personal information and COVID-19 infection status through a questionnaire. The risk perception of residents was analyzed after adjusting the zero COVID policy, and the factors influencing COVID-19 risk perception were explored. Our research findings can contribute to the development of a solid research foundation for the prevention and management of COVID-19 outbreaks among Beijing residents and in other regions. In this paper, we ask two crucial questions about COVID-19; (a) how concerned are people? and (b) what social-psychological factors determine their level of concern?

## 2 Materials and methods

### 2.1 Procedure

This study was a cross-sectional study aiming to investigate the health situation of community residents during the COVID-19 pandemic and the impact of the coronavirus disease, conducted by a research team from Peking University Third Hospital and School of Public Health, Peking University. The sample of our study is community residents who have experienced the Omicron outbreak from late 2022 to early 2023, since the Chinese government declared the “new 10 prevention and control measures” and ended the zero-COVID strategy in December 2022. Data collection took place between January 13th and February 13th, 2023.

### 2.2 Participants

To ensure the representativeness of the survey sample, this study used the 16 administrative districts of Beijing as the basic sampling frame and randomly selected 8 to 42 Community Healthcare Centers in each district based on population proportion, totaling 293 Community Healthcare Centers. Within each center, 3 to 5 family-contracted doctors were selected, and each doctor selected 40 to 50 contracted residents as the survey subjects. The study conducted a questionnaire survey on a total of 60,039 residents. 5,624 responses were excluded because they were not filled out properly (missing essential information). This resulted in a final sample of 54,415 participants.

### 2.3 Measures

Following Xie et al. (2005), van der Linden (2015), and Dryhurst et al. (2020), our dependent variable “COVID-19 Risk Perception” was measured as a set of indexes, involved with several dimensions to provide a comprehensive measure of RP.

### 2.4 Predictors

Our social-psychological predictor variables were based on the “climate change risk perception model” (CCRPM) by van der Linden (2015), which included measurements of personal experience and socio-demographics (Table 2). Specifically, we included items on direct contagious experience with the SARS-CoV-2 virus, as well as income, career, payment type, and chronic medical history.

## 3 Results

We collected basic information about the citizens, most of whom live in Beijing (Table 1). With 5,624 responses eliminated due to absent information, 54,415 samples were included in our analysis. About 64% of them were female and 84% had got infected with COVID-19 at least once.

**Table 1:** Basic information about the citizens in the study.

Characteristic	*N* = 54,415
*Gender*	
Male	19,646 (36%)
Female	34,769 (64%)
*Age*	
18 ~ 30	8,122 (15%)
31 ~ 45	20,362 (37%)
46 ~ 60	16,384 (30%)
>60	9,547 (18%)
*Marriage*	
Married	46,328 (85%)
Others	8,087 (15%)
*Education level*	
Middle school and below	10,720 (20%)
High school/Technical school	25,005 (46%)
College degree and above	18,690 (34%)
*Region*	
Central city	14,548 (27%)
Suburb	39,867 (73%)
*Income*	
Rich	11,768 (22%)
Ordinary	41,446 (76%)
Poor	1,201 (2%)
*Healthcare worker*	
Y	7,129 (13%)
N	47,286 (87%)
*Payment type*	
Urban employee medical insurance	40,007 (74%)
Urban and rural resident medical insurance	8,293 (15%)
Free medical insurance	2,505 (5%)
Uninsured	2,762 (5%)
Other	848 (2%)
Live alone	
Y	2,611 (5%)
N	51,804 (95%)
*Chronic disease*	
Y	35,455 (65%)
N	18,960 (35%)
*Infected with Covid-19*	
Y	45,451 (84%)
N	8,964 (16%)

n (%).

### 3.1 Exploratory factor analysis

After that, we conducted the exploratory factor analysis (EFA) for the dimensionality reduction analysis of the scale. First, the Kaiser–Meyer–Olkin (KMO) test and Bartlett test of sphericity were implemented to test the suitability of the data. KMO was calculated as 0.9 (≥ 0.9); besides, EFA was validated to be suitable by the Bartlett test of sphericity (*χ*^2^: 430.3598, *p* < 0.001). Then, we successfully performed EFA on the data, as such, factors were extracted through varimax rotation. According to Table 2, all 11 items were divided into three dimensions, accounting for 53% variance. Drawing on insights from psychological theory and literature (Qin et al., 2021), dimensions 1 to 3 were distinctly classified as “Perceived health threat,” “Perceived severity & controllability,” and “Perceived infection possibility.”

**Table 2:** Exploratory factor analysis of the risk perception scale.

Risk perception scale	Component		
	Factor 1	Factor 2	Factor 3
1. The degree of worry about COVID-19	−0.08	−0.06	0.90
(not worried at all-very worried)			
2. The impact of COVID-19 on individuals	0.00	0.00	0.79
(small-large)			
3. The impact of COVID-19 on society	0.15	0.10	0.56
(small-large)			
4. The consequences of COVID-19	0.43	0.06	0.12
(delayed-immediate)			
5. The characteristics of COVID-19	0.62	−0.05	0.06
(natural-artificial)			
6. The impact of infection with SARS-CoV-2	0.61	0.11	0.10
(short term-long term)			
7. Perceived society’s control of COVID-19	0.66	0.18	−0.10
(controllable-uncontrollable)			
8. Perceived individuals’ control of COVID-19	0.16	0.65	−0.01
(evitable-inevitable)			
9. The knowledge about COVID-19	0.54	−0.09	−0.05
(familiar-unfamiliar)			
10. Perceived probability of infection with COVID-19	−0.04	0.83	0.03
among the general population (small-large)			
11. Perceived probability that I get infected with	−0.11	0.93	−0.04
COVID-19 (small-large)			

### 3.2 Relations between dimensions of risk perception

By analyzing the relations between the dimensions of risk perception, we would have a better understanding of the influencing factors of COVID-19-related worries. As such, we ran a simultaneous regression analysis, using the degree of COVID-19-related worries as dependent variables, and other dimensions of RP as predictors (Table 3). Our indicators of all dimensions, in fact, were significantly associated with COVID-19-related worries, except for perceived society’s control of the epidemic. People who perceived more probability of infection tend to have less worry about COVID-19 (β = −0.05, [95% CI; −0.06, −0.04]), suggesting that individuals who are more exposed to information about the virus or have experienced COVID-19 firsthand could develop a sense of familiarity or desensitization to the risks, leading to decreased anxiety levels.

**Table 3:** Analysis of the relations in risk characteristics of COVID-19.

Characteristic	β	95% CI1	*p*-value
The impact of COVID-19 on individuals	0.47	0.46, 0.48	<0.001
The impact of COVID-19 on society	0.28	0.27, 0.29	<0.001
The consequences of COVID-19	0.02	0.01, 0.03	<0.001
The characteristics of COVID-19	0.04	0.03, 0.05	<0.001
The impact of infection with SARS-CoV-2	0.05	0.04, 0.06	<0.001
Perceived society’s control of COVID-19	0.00	−0.01, 0.01	0.55
Perceived individuals’ control of COVID-19	0.05	0.04, 0.06	<0.001
The knowledge about COVID-19	0.02	0.01, 0.03	<0.001
Perceived probability of infection with COVID-19	0.04	0.03, 0.05	<0.001
among the general population			
Perceived probability that I get infected with	−0.05	−0.06, −0.04	<0.001
COVID-19			

Research suggests that the dimension of whether a risk event is “not at all worried” or “very worried” has been considered a significant contributor to the degree of risks. The results of our study reconfirmed this assumption, in part, it is obvious that the “very worried” experience is directly related to psychological panic.

### 3.3 Determinants of COVID-19 risk perception

Next, we ran a multiple linear regression model to illustrate a detailed overview of the predictors that make a difference in COVID-19 risk perception (Table 4). Based on the results of EFA, our indicators, within the predictor model, of experience with the virus, history of chronic diseases, payment types, as well as socio-demographics, were all significantly related to three dimensions of risk perception.

**Table 4:** Regression outputs of the risk perception of COVID-19 among citizens.

Experiential and socio-demographic factors	Perceived health threat		Perceived severity and controllability		Perceived infection possibility	
	β^a^	95% CI	β^a^	95% CI	β^a^	95% CI
*Gender*						
Male	—	—	—	—	—	—
Female	1.8^***^	1.7, 1.9	1.1^***^	1.0, 1.2	0.92^***^	0.84, 1.0
*Age*						
18 ~ 30	—	—	—	—	—	—
31 ~ 45	1.1^***^	0.90, 1.2	0.81^***^	0.63, 1.0	1.1^***^	1.0, 1.3
46 ~ 60	1.4^***^	1.2, 1.6	0.67^***^	0.47, 0.86	1.3^***^	1.1, 1.4
>60	1.5^***^	1.3, 1.7	1.4^***^	1.1, 1.6	1.3^***^	1.1, 1.5
*Marriage*						
Married	—	—	—	—	—	—
Others	−0.41^***^	−0.58, −0.25	−0.07	−0.24, 0.09	−0.23^**^	−0.36, −0.09
*Education level*						
Middle school and below	—	—	—	—	—	—
High school/Technical school	0.18^*^	0.04, 0.32	0.30^***^	0.16, 0.45	0.46^***^	0.34, 0.57
College degree and above	0.19^*^	0.02, 0.36	0.92^***^	0.75, 1.1	1.3^***^	1.1, 1.4
*Region*						
Central city	—	—	—	—	—	—
Suburb	0.07	−0.05, 0.18	−0.56^***^	−0.68, −0.45	−0.16^***^	−0.26, −0.07
*Income*						
Rich	—	—	—	—	—	—
Ordinary	−1.7^***^	−1.8, −1.6	−1.5^***^	−1.6, −1.4	−1.0^***^	−1.1, −0.86
Poor	−3.0^***^	−3.4, −2.7	−2.6^***^	−3.0, −2.3	−1.9^***^	−2.2, −1.6
*Healthcare worker*						
Y	—	—	—	—	—	—
N	0.77^***^	0.62, 0.92	1.1^***^	0.91, 1.2	−0.16^*^	−0.28, −0.03
*Payment type*						
Urban employee medical insurance	—	—	—	—	—	—
Urban and rural resident	−0.37^***^	−0.52, −0.22	−0.59^***^	−0.74, −0.44	−0.60^***^	−0.72, −0.47
Medical insurance						
Free medical insurance	−0.12	−0.36, 0.11	−0.39^**^	−0.63, −0.16	−0.28^**^	−0.47, −0.09
Uninsured	−0.41^***^	−0.64, −0.18	−0.47^***^	−0.70, −0.24	−0.64^***^	−0.83, −0.46
Other	−0.11	−0.51, 0.28	0.07	−0.33, 0.46	0.00	−0.32, 0.32
*Live alone*						
Y	—	—	—	—	—	—
N	0.62^***^	0.38, 0.86	0.37^**^	0.13, 0.61	0.40^***^	0.21, 0.59
*Chronic disease*						
Y	—	—	—	—	—	—
N	1.4^***^	1.2, 1.5	1.1^***^	0.94, 1.2	0.83^***^	0.74, 0.92
*Infected with Covid-19*						
Y	—	—	—	—	—	—
N	0.60^***^	0.46, 0.73	0.07	−0.07, 0.20	−1.6^***^	−1.7, −1.5

a Multiple linear regression.

*p < 0.05, **p < 0.01, ***p < 0.001.

Specifically, there is a gender effect, such that females perceive more risk compared to males (e.g., β = 1.8, [95% CI; 1.7, 1.9]). Aging plays a considerable role in contributing to increased concerns about COVID-19, in part, individuals over 60 perceive more risk versus youngsters aged 18 to 30 (e.g., β = 1.5, [95% CI; 1.3, 1.7]). The less money people think they earn compared to others around, the less risk they perceive (e.g., β = −3.0, [95% CI; −3.4, −2.7]), in other words, the relative level of income is negatively associated with RP. Results show that the currently married (e.g., β = 0.41, [95% CI; 0.25, 0.58]) and residents of the central city (e.g., β = 0.56, [95% CI; 0.45, 0.68]) perceive more risk, while healthcare workers (e.g., β = −1.1, [95% CI; −1.2, −0.91]) and people who live alone (e.g., β = −0.62, [95% CI; −0.86, −0.38]) appear fewer concerns about COVID-19. Educational level is positively correlated with risk perception, in addition to a payment effect, such that residents without medical insurance perceive less risk than those covered by Urban Employee Medical Insurance (e.g., β = −0.64, [95% CI; −0.83, −0.46]). People who have had contagious experience with the virus perceive less threat (β = −0.60, [95% CI; −0.73, −0.46]), but more infection possibilities (β = 1.6, [95% CI; 1.5, 1.7]) in comparison with those who have never been infected yet. Besides, there is a negative correlation observed between the history of chronic diseases and the factors of RP (e.g., β = −1.4, [95% CI; −1.5, −1.2]).

## 4 Discussion

In this paper, we set out to analyze and model the risk perception of COVID-19 mostly in Beijing. Across the study, we find that worries about COVID-19 are significantly influenced by other dimensions of RP. We divided 11 items of RP into three general dimensions, based on the exploratory factor analysis which we have conducted. Several socio-psychological factors emerged as critical predictors. Consistent with the achievements in the domain of contagious risk, experiential, and socio-demographic factors show important correlations in our RP regression model.

The results provide a comprehensive understanding of the sources of psychological anxiety in the population, which involves the structure of risk perceptions. Even though this may not reflect the overall pattern of people’s perceptions of risk events and hazards, for the specific risk of COVID-19 pandemics, however, the perceived society’s control of the epidemic has not played a major role in RP. Concerns and panics hardly originate from the social response system and policy of COVID-19, instead, it is probably the pandemic itself that matters to the residents. Our study also reveals that individuals with higher levels of education and income tended to have higher risk perceptions of COVID-19. This could be attributed to their greater access to information and resources, allowing them to better understand the potential risks and consequences of the SARS-CoV-2 virus (Honarvar et al., 2020). In addition, we find that overall risk perceptions of COVID-19 in Beijing have maintained a relatively moderate level (44 out of 77 on average).

A possible explanation is that the capital city of China has experienced an unstoppable outbreak of the Omicron variant (Dyer, 2022), after which 84 percent of the residents have got infected by the virus at least once. While COVID-19 can hit twice and even more, it is not likely to experience reinfections within 3 months after the first diagnosis of infection (Pilz et al., 2022), and this knowledge may have reduced the public’s awareness and concern. Interestingly, results show that risk perceptions varied across different age groups, with older individuals perceiving a higher risk than younger individuals. This may be due to the fact that older individuals are more vulnerable to severe complications and even death from COVID-19. On the other hand, retired seniors may struggle to perceive support from organizations in the same way younger workers do, which is negatively correlated with the social dimension of well-being (Capone et al., 2022).

In short, our findings considerably suggest that risk perceptions of COVID-19 in Beijing are influenced by a variety of socio-experiential factors, including age, gender, education, and income. These findings can contribute to public health efforts and strategies aimed at promoting greater awareness and governance of infectious diseases. We provide valuable insights into the determinants of COVID-19-related worries and risk perception among the residents in Beijing, most notably the “big picture” overview of communication between human and natural hazards. By analyzing these factors, we can pave the way for rewarding health campaigns and patient education, ultimately building trust and encouraging health-protective behaviors. The importance of mental well-being cannot be overstated, as it is significantly linked to our overall health and resilience, affecting our capacity to cope with challenges across the lifespan.

And of course, our research is not without limitations. The sample size and demographics of the participants may have influenced the results, and further research is needed to confirm the validity of these findings in other settings. It is important to notice that although our samples were abundant in number, they were residents who have been to the medical institutions and therefore are not completely representative of the population in Beijing. In addition, we conducted a massive cross-sectional study that covered different aspects of COVID-19, in other words, it is not specific research only focused on risk perception. As such, our study was not exhaustive, and other significant factors ought to be considered, including Non-Technical Skills (Converso et al., 2021).

## 5 Conclusions and implications

This study evaluates the specific content and structure of COVID-19-related risk perceptions, as well as their determinants. It is essential to understand the risk perceptions and health-protective behaviors of residents with diverse educational levels, incomes, and medical histories. By doing so, we would develop targeted interventions to address the specific concerns and needs of different population groups and ultimately contribute to the effective prevention and control of infectious diseases.

## Data availability statement

The raw data supporting the conclusions of this article will be made available by the authors, without undue reservation.

## Author contributions

QL: Conceptualization, Data curation, Investigation, Writing – review & editing. YT: Methodology, Visualization, Writing – original draft. ZZ: Formal analysis, Methodology, Software, Writing – review & editing. JZ: Conceptualization, Investigation, Resources, Writing – review & editing. YF: Writing – review & editing. QW: Writing – review & editing. ZN: Writing – review & editing. LY: Funding acquisition, Supervision, Writing – review & editing, Investigation. XL: Supervision, Project administration, Resources.
